# Phylogenetic analysis and antimicrobial susceptibility profile of uropathogens

**DOI:** 10.1371/journal.pone.0262952

**Published:** 2022-01-28

**Authors:** Hanif Ullah, Kashif Bashir, Muhammad Idrees, Amin Ullah, Neelma Hassan, Sara Khan, Bilal Nasir, Tariq Nadeem, Hina Ahsan, Muhammad Islam Khan, Qurban Ali, Sher Muhammad, Muhammad Afzal

**Affiliations:** 1 Department of Health & Biological Sciences, Faculty of life Sciences, Abasyn University, Peshawar, Pakistan; 2 Department of Biotechnology, University of Swabi, Swabi, Pakistan; 3 Center of Excellence in Molecular Biology, University of The Punjab, Lahore, Pakistan; 4 Faculty of Pharmacy, Riphah International University, Islamabad, Pakistan; 5 Institute of Molecular Biology and Biotechnology, University of Lahore, Lahore, Pakistan; 6 Department of Bioinformatics and Biotechnology, Government College University Faisalabad, Faisalabad, Pakistan; Government College University Faisalabad, PAKISTAN

## Abstract

The uropathogens is the main cause of urinary tract infection (UTI). The aim of the study was to isolate bacteria from urine samples of UTI patients and find out the susceptibility of isolated bacteria. Bacteria were identified using both conventional and molecular methods. Sanger sequence procedure used for 16S ribosomal RNA and phylogenetic analysis was performed using Molecular Evolutionary Genetics Analysis (MEGA-7) software. In this study, *Escherichia coli*, *Klebsiella pneumonia*, *Staphylococcus* were reported as 58, 28 and 14.0% respectively. Phylogenetic tree revealed that 99% of sample No. Ai (05) is closely related to *E*. *coli* to (NR 114042.1 *E*. *coli* strain NBRC 102203). Aii (23) is 99% similar to *K*. *pneumoniae* to (NR 117686.1 *K*. *pneumonia* strain DSM 30104) and 90% Bi (48) is closely linked to *S*. *aureus* to (NR 113956.1 *S*. *aureus* strain NBRC 100910). The antibiotic susceptibility of *E*. *coli* recorded highest resistance towards ampicillin (90%) and least resistant to ofloxacin (14%). Some of the other antibiotics such amoxicillin, ciprofloxacin, gentamicin, ceftazidime, cefuroxime and nitrofurantoin resistance were observed 86, 62, 24, 55, 48 and 35% respectively. The cefuroxime showed the highest antibiotic resistance against *K*. *pneumoniae* with 85% followed by amoxicillin, ciprofloxacin, gentamicin, ceftazidime, ampicillin and nitrofurantoin resulted in 60, 45, 67, 70, 75 and 30% respectively. The resistance of *S*. *aureus* against erythromycin, cefuroxime and ampicillin were found with 72%. The resistance against amoxicillin, gentamicin, ceftazidime and ceftriaxone found 57, 43, 43 and 15% respectively. Phylogenetic analysis shows that sequences are closely related with the reference sequences and *E*. *coli* is the dominant bacteria among UTI patients and is resistant to the commercially available antibiotics.

## Introduction

The most common infection caused by microbes is urinary tract infection. It occurs in the people belonging to all age groups, in which *Escherichia coli* has significant etiologic factor ranging from 50–80 percent of cases. Strains isolate from UTI, like *E*. *coli* are known as uropathogenic [1, 2]. Among human, the most common bacteria that causes UTI is *E*. *coli*, *K*. *pneumoniae*, *E*. *faecalis*, *S*. *marcescens*, *P*. *aeruginosa*, *S*. *aureus S*. *saprophyticus* and *Proteus mirabilis* [3]. The prevalence of UTI in pediatrics has been high in Asian countries. The frequency of UTI reported in Nepal at different time from different areas that were 15.88% and 57% [4]. Another study conducted in India, 48% prevalence shown [5] and in Nigeria, the incidence of UTI among adolescents and children was 11.9% [6]. Study in Kenya on the contribution of UTI to the burden of febrile diseases in young children reported a proportion of approximately 11.9% [7]. Different studies are conducted in different research groups in Ethiopia. The results among pediatric patients at referral hospital of Hawassa showed that the UTI prevalence was 27.5% [8]. UTI is a major concern for women in particular; almost 50% of women get at least one UTI in their lifespan and about 25% of those will have one or more repeated infections [9]. UTI status has been not described in Mongolia, but 60.3% of all patients with chronic pyelonephritis were females between 20 and 40 years of age [10]. The main cause of community-acquired UTIs (70–95%) and a large portion of nosocomial UTIs (50%) are strains of uropathogenic *Escherichia coli* (UPEC), responsible for major medical costs and morbidity worldwide [11].

*Escherichia coli* is the main etiological agent responsible for all UTIs with 70–90% [12]. Microbes create such situation in which they can damage healthy body in time through gene transfer mechanisms and supported as the amount of pathogenicity-associated islands [13]. In microbial ecology, identification of bacterial species widely used to determine the biodiversity of environmental samples and to diagnose infected patients through medical microbiology. Bacteria can be classified using conventional methods of microbiology, such as microscopy, specific media growth, biochemical and serological testing, and assays of antibiotic sensitivity. Molecular methods of microbiology have revolutionized bacterial identification in recent decades. 16S (rRNA) gene sequencing is a popular method. This method is not only faster and more accurate than conventional methods, but also enables to identify the strains which are difficult to grow under laboratory conditions. In addition, strain differentiation at the molecular level allows discrimination between phenotypically identical bacteria [14].

Molecular and genetic research approaches have been used mostly for phylogenetic relationships between various bacteria using different genomic regions. The comparative analysis of different rRNA genes are routinely used to study the phylogenetic diversity of different bacterial species due to their highly conserved nature and ease of amplification [15]. The 16S rRNA gene sequence comparison, however, has become a gold standard genetic technique for differentiating different species, identifying unknown bacteria, and comparing the genetic relationship between isolates, thus grouping closely related organisms into clonal complexes [16]. UTI and antibiotic resistance is a major concern and less knowledge is available in the studied population therefore, this study was planned to conduce.

## Materials and methods

### Sample collection

The study population was drawn from patients suspected for UTI coming to Lady Reading Hospital and private clinics / laboratories. A total of fifty early morning mid-stream urine specimens were collected in sterile, dry, wide-mouth leak proof containers. The samples were labelled and instantly transported to the microbiology research laboratory at Abasyn University, Peshawar, for further processing. The authors have confirmed that this specific study was reviewed and approved by Abasyn University, Peshawar review board (ethics committee) before the study began. The written consent was taken which was documented and witnessed by Abasyn University, Peshawar review board (ethics committee).

### Inclusion and exclusion criteria

Male and female of all age, ethnic group and races were included in this study, in addition, people who were diagnosed with UTI in real time PCR laboratory while people not having UTI or with other renal problem and infection were excluded from the study.

### Macroscopic/ microscopic examination of urine

Urine samples were physically examined by swirling or inverting the bottles for the presence or absence of cloudiness or turbidity. Turbidity indicated the presence of bacteria, proteins, crystals, leucocytes, precipitation of urates (acids) or phosphates and carbonates (alkaline).

### Culture media used

Different media were used for the culturing of samples i.e. Nutrient Agar, MacConkey Agar Media and Cysteine Lactose Electrolyte Deficient (CLED) Agar [17].

### Identification of isolates

To identify uropathogens morphologically and biochemically different identification tests were used for their isolation. Colonies were identified after incubation at 37°C for 24–48 hours using Gram staining, Biochemical Identification [18].

### Molecular identification

Pure bacteria culture of nutrient agar was sent to Macrogen Company Korea for 16S rRNA sequencing. The data were analyzed by using Finch TV software, version 1.4.0 (www.geospiza.com) [19]. Codon-code aligner version 9.0.1 was used to obtain contig sequences [20]. The contig sequences were compared to the reference 16S rRNA sequences at National Center for Biotechnology Information (NCBI) database by using Basic Local Alignment Search Tool (BLAST) algorithm. Phylogenetic tree was constructed based on the nucleotide sequences by using Molecular Evolutionary Genetic Analysis (MEGA-7), version 10.2.2 (Build No. 10201106-x86_64) software [21].

### Antibiotic susceptibility testing

Sensitivity test was done by the Rule of modified Kirby-Bauer disk diffusion method and Clinical laboratory standard institute (CLSI) guidelines. According to the procedure followed by Panthi *et al*., [22]. Discs containing the following antibacterial agents were used: Amoxicillin/Clavulanate, Ciprofloxacin, Gentamicin, Cefuroxime, Ceftazidime, and Ampicillin, Nitrofurantoin against Gram negative bacteria and Amoxicillin/Clavulanate, Cloxacillin, Erythromycin, Ceftriaxone, Gentamicin, Cefuroxime, Ceftazidime against gram positive bacteria.

#### Kirby Bauer disk diffusion method

The bacteria were collected having identical morphology with almost 3 isolated colonies. Mannitol Salt Agar (MHA) were prepared for discs diffusion method. It is then autoclaved for 15 min at 121°C. MHA media poured into sterilized petri plates. Then all isolated colonies were picked up by sterile swab from fresh culture and streaked on MHA plates for making more than one colony. Different antibiotics discs were used for checking susceptibility. Specific antibiotic discs placed through sterile forceps for each specific species of culture at same distance. Finally, the plates incubated for 24 hours at 37°C and zones of inhibition were measured in millimeter. According to the CLSI, 2020, the interpretative chart which derived from the zones of inhibition of standard organisms were used and the zone size of each antimicrobial agent was interpreted.

## Results

In this study, totally 50 urine positive samples were obtained from Lady Reading Hospital (LRH) and private clinics / laboratory at district Peshawar, Khyber Pakhtunkhwa (KP) Pakistan. Samples were collected from adult male and female and processed for identification on the basis of gram staining, colony morphology and biochemical test.

### Percentage on the basis of bacterial isolated species

**Fig 1:**
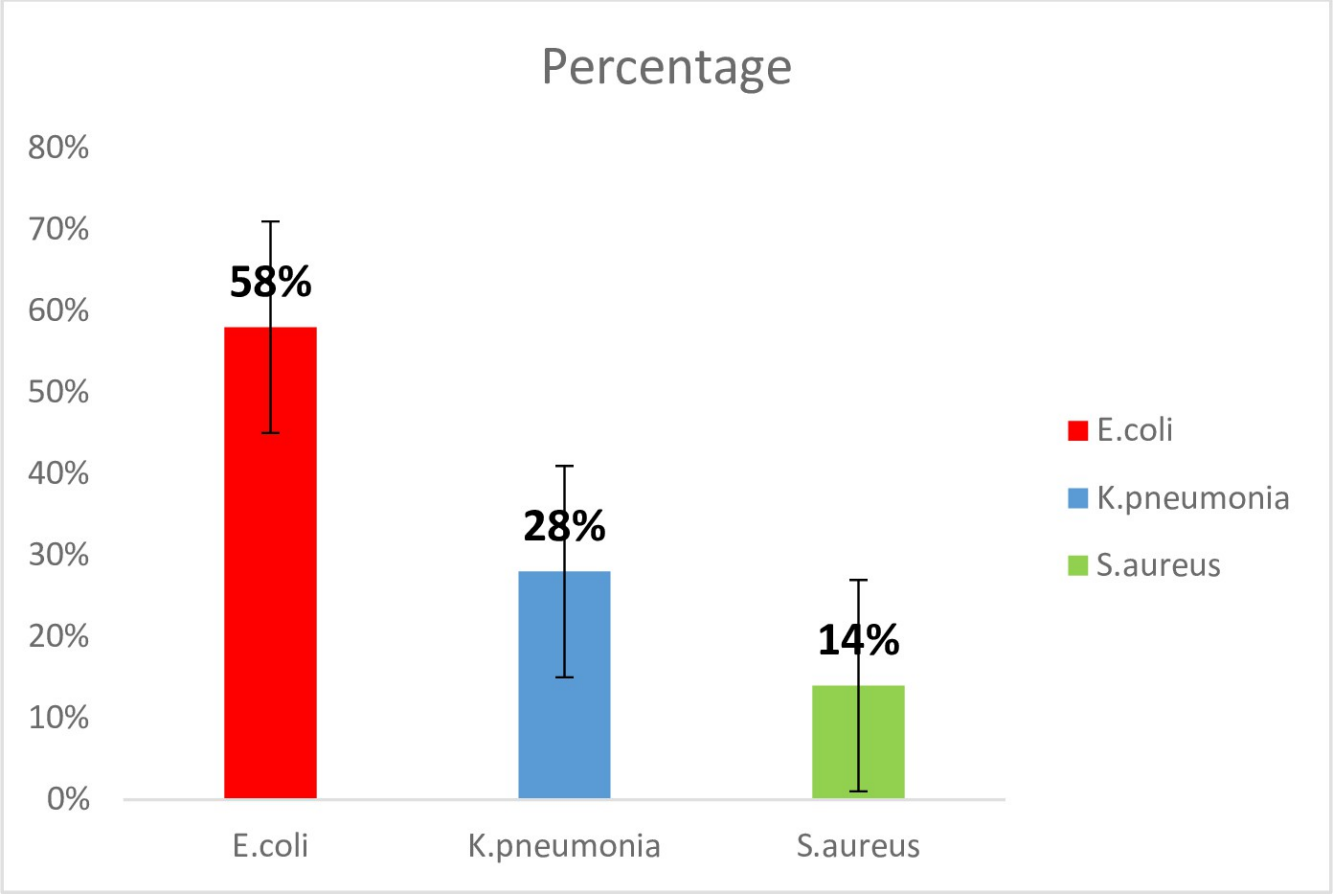
Percentage of bacterial species isolated from urine samples.

There were 50 samples of patients suffering from UTI, which showed *E*.*coli* (58.0%), *K*. *pneumoniae (28*.*0%)*, and *S*. *aureus (*14.0%) as shown in Fig 1.

#### Prevalence on the basis of gender

**Fig 2:**
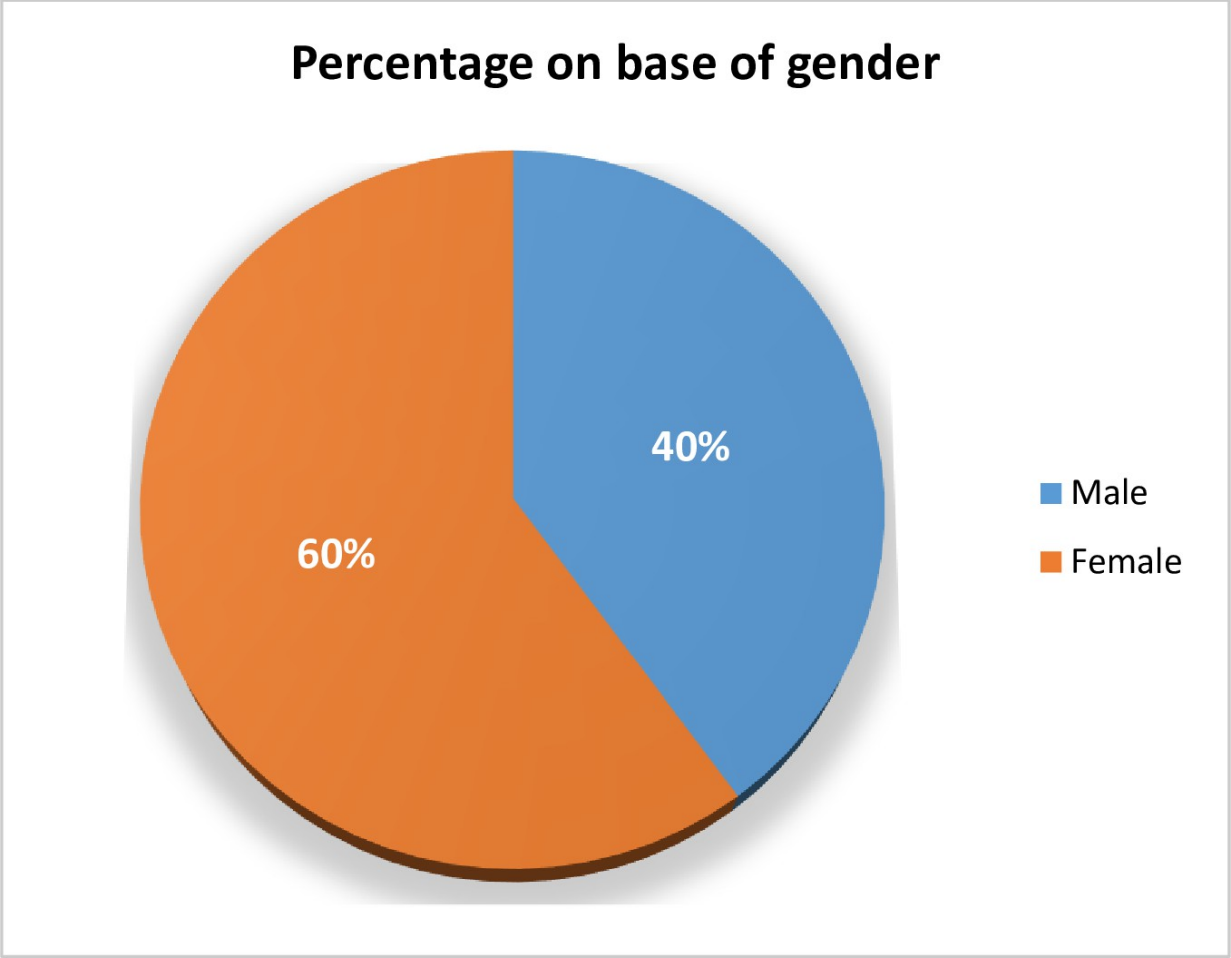
Gender base distribution of positive isolates.

Total 20 positive isolates were recovered from male while 30 urine specimens were positive from female patients as shown in Fig 2.

#### Colony characteristics of Isolated Bacteria on Cystine Lactose-Electrolyte-Deficient (CLED) agar media

In our data, three bacterial isolates were observed with different colony characteristic. *E*. *coli* observed with yellow opaque colonies with deeper yellow center, *K*. *pneumoniae* with yellow to whitish blue colonies very mucoid and *S*. *aureus* with pale to deep yellow colonies shown in Table 1.

**Table 1:** Colony characteristic on CLED agar media.

Bacteria	Colony characteristics
*E*.*coli*	A yellow Opaque colonies with a slightly deeper yellow center
*K*. *pneumoniae*	Yellow to whitish blue colonies very mucoid
*S*. *aureus*	Yellow Pale to deep yellow colonies.

Further these isolates were identified by different biochemical test such as oxidase, lactose fermentation, indole, ureases, and citrate test for gram negative bacteria and catalase test and mannitol fermentation for gram positive bacteria through bergey’s manual identification flow charts. The results are summarized in Table 2.

**Table 2:** Identification of gram negative and gram positive bacteria on basis of biochemical test.

No. of isolates	Bacterial isolates	Gram staining	Oxidase test	MacConkey agar	Indole test	Ureases test	Citrate test	Catalase test	Mannitol fermentation
29	*E*.*coli*	- ve	_	LF	+	NA	_	NA	NA
14	*K*. *pneumoniae*	- ve	_	LF	_	NA	NA	NA	NA
07	*S*. *aureus*	+ ve	NA	NA	NA	NA	NA	+	+

**Symbol**: (–) negative, (LF) lactose fermentation, (NA) (+) positive, (NA) not applicable.

### Molecular identification of isolated bacteria of urine samples

Among the 50 isolated samples, three different bacterial species were obtained (*E*. *coli*, *K*. *pneumonia*, and *S*. *aureus*). From these isolates samples No. Ai (05), Aii (23) and Bi (48) were further evaluated for 16S rRNA sequencing through Macrogen, Korea. The results were submitted to NCBI. After processing, accession numbers were allotted to the samples as given in Table 3.

**Table 3:** NCBI Accession number strain abbreviation and other description of three isolates.

S. No.	Description	Strain Abbreviation	NCBI Accession No.
1.	*E*. *coli*	Ai (05)	MW451080
2.	*K*. *pneumoniae*	Aii (23)	MW453025
3.	*S*. *aureus*	Bi (48)	MW453043

**Fig 3:**
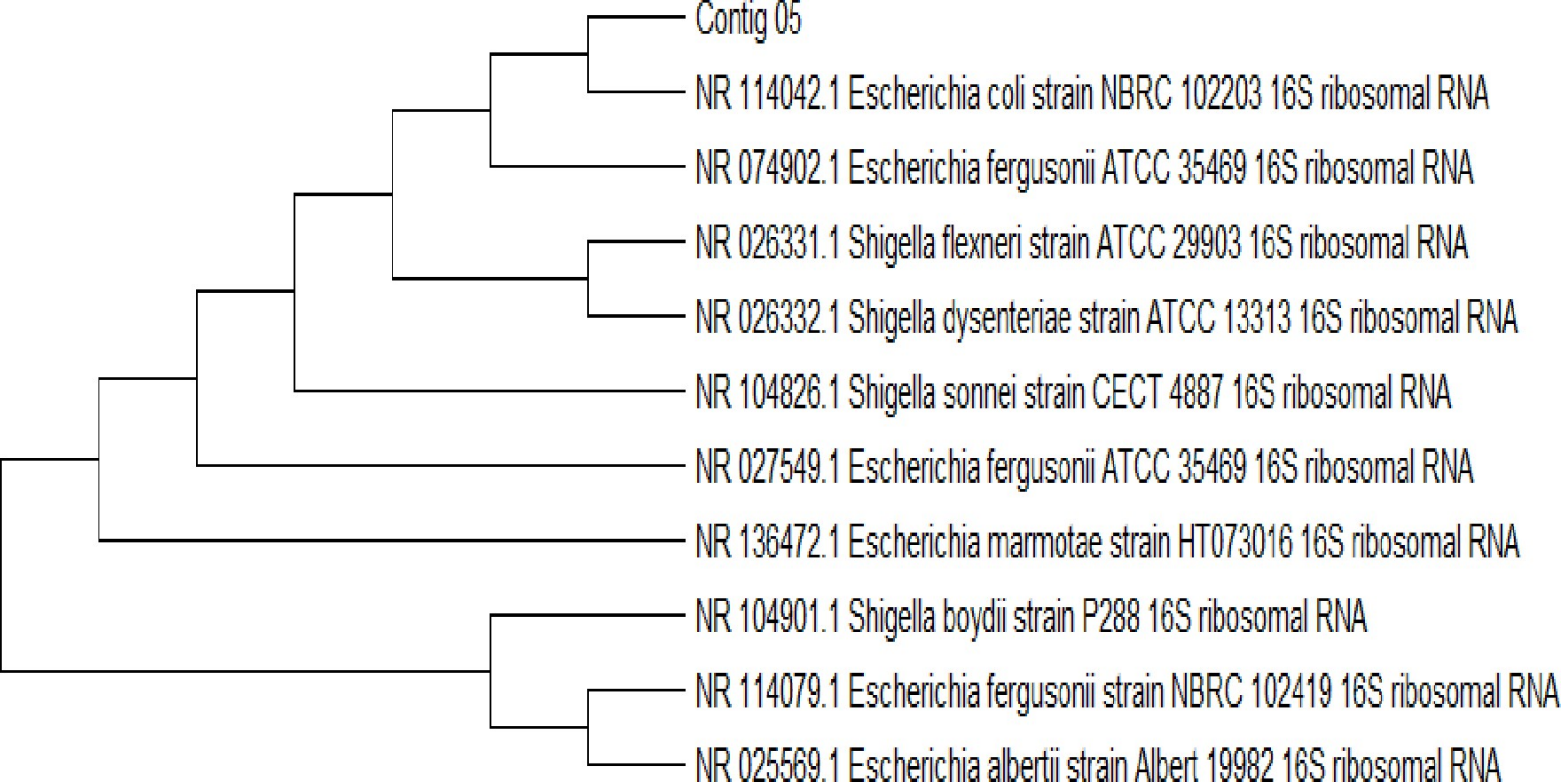
Phylogenetic tree of *E*. *coli* (Ai 05).

**Fig 4:**
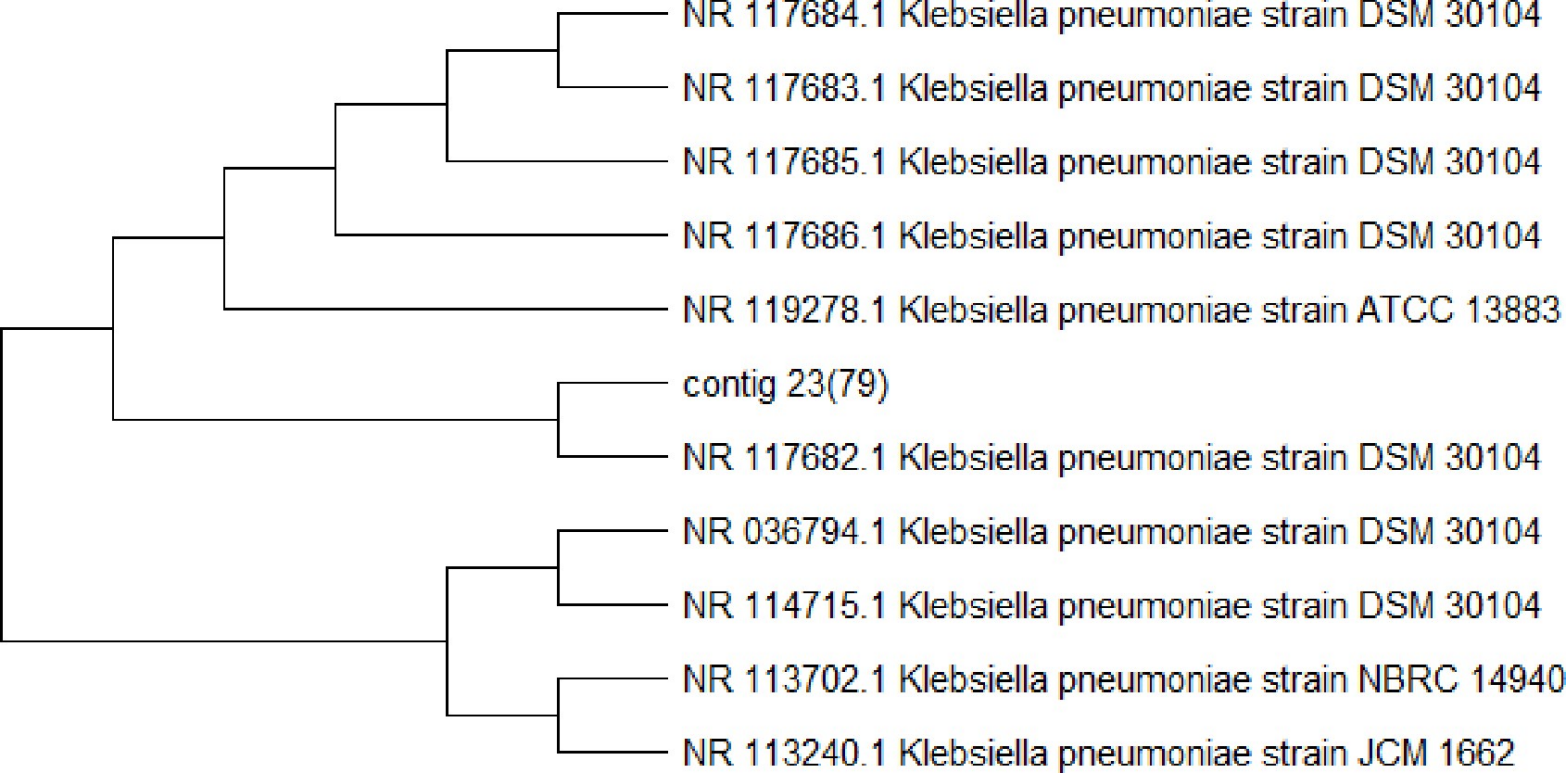
Phylogenetic tree of *K*. *pneumoniae* (Aii, 23).

**Fig 5:**
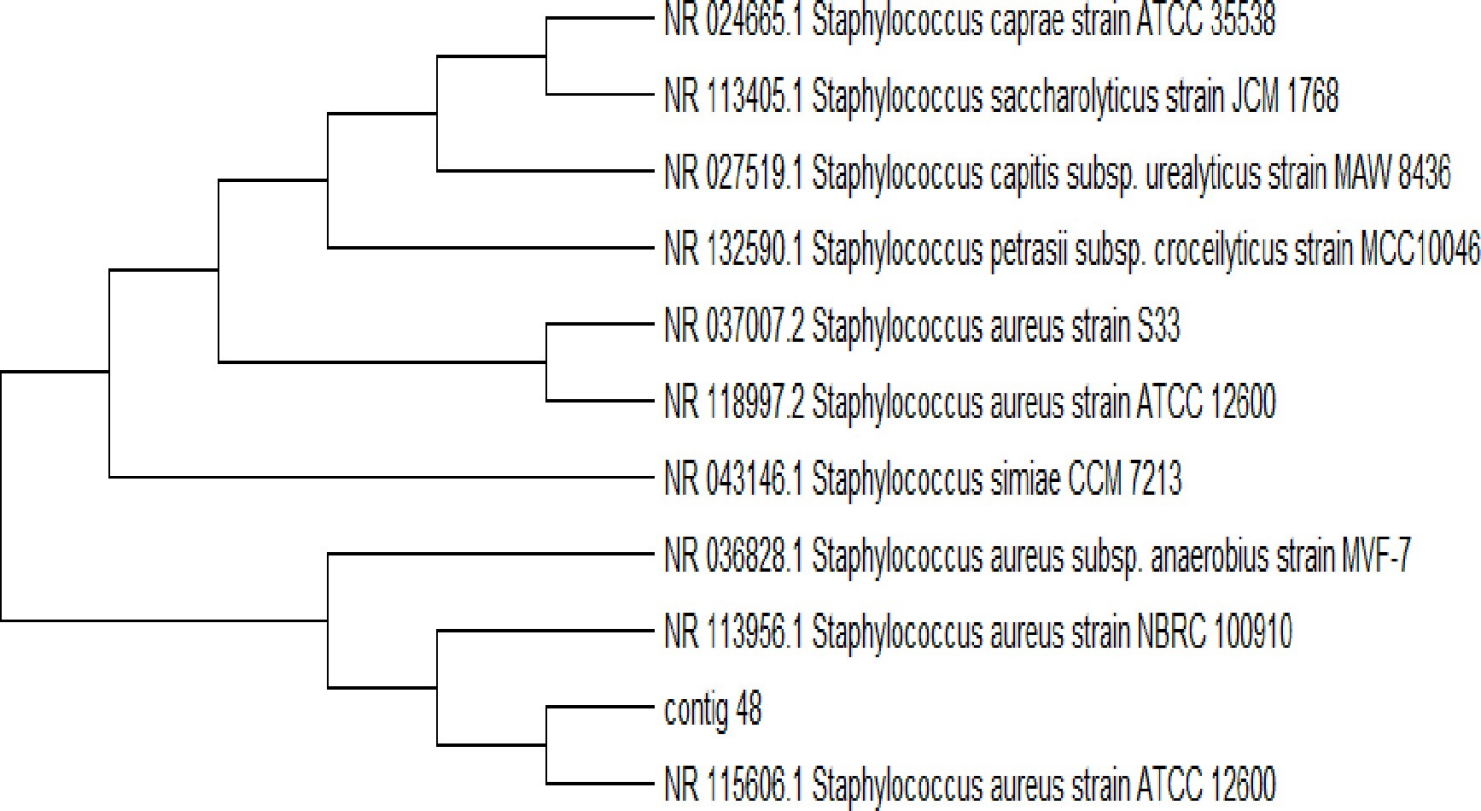
Phylogenetic tree of *S*. *aureus* (Bi, 48).

Subsequently, phylogenetic tree were made with similar sequences by using neighbor joining method for each isolated bacterial strain (Figs 3–5). The phylogenetic tree showed that Ai (05) is 99% related to (NR 114042.1 *E*.*coli* strain NBRC 102203). Aii (23) is 99% similar to (NR 117686.1 *K*. *pneumonia* strain DSM 30104). Bi (48) is 90% related to (NR 113956.1 *S*. *aureus* strain NBRC 100910).

The 16S rRNA sequences of Ai (05) (NR 114042.1 *E*. *coli* strain NBRC 102203), Aii (23) (NR 117686.1 *K*. *pneumonia* strain DSM 30104) and Bi (48) (NR 113956.1 *S*. *aureus* strain NBRC 100910), were submitted to NCBI (https://submit.ncbi.nlm.nih.gov/subs/genbank/) for the allotment of accession number through web portal of NCBI. NCBI issued Accession numbers to the submitted sequences after proper processing as given in the Table 4.

**Table 4:** The E. value, similarity and other description of three isolates.

Description	Strain abbreviation	Query cover (%)	E. value	Identity (%)	Accession No.
*E*. *coli*	Ai (05)	99%	0.0	99%	MW451080
*K*. *pneumonia*	Aii (23)	99%	0.0	99.59%	MW453025
*S*. *aureus*	Bi (48)	96%	0.0	90%	MW453043

#### Susceptibility pattern of isolated bacteria (*E*. *coli)* from UTI patients

**Fig 6:**
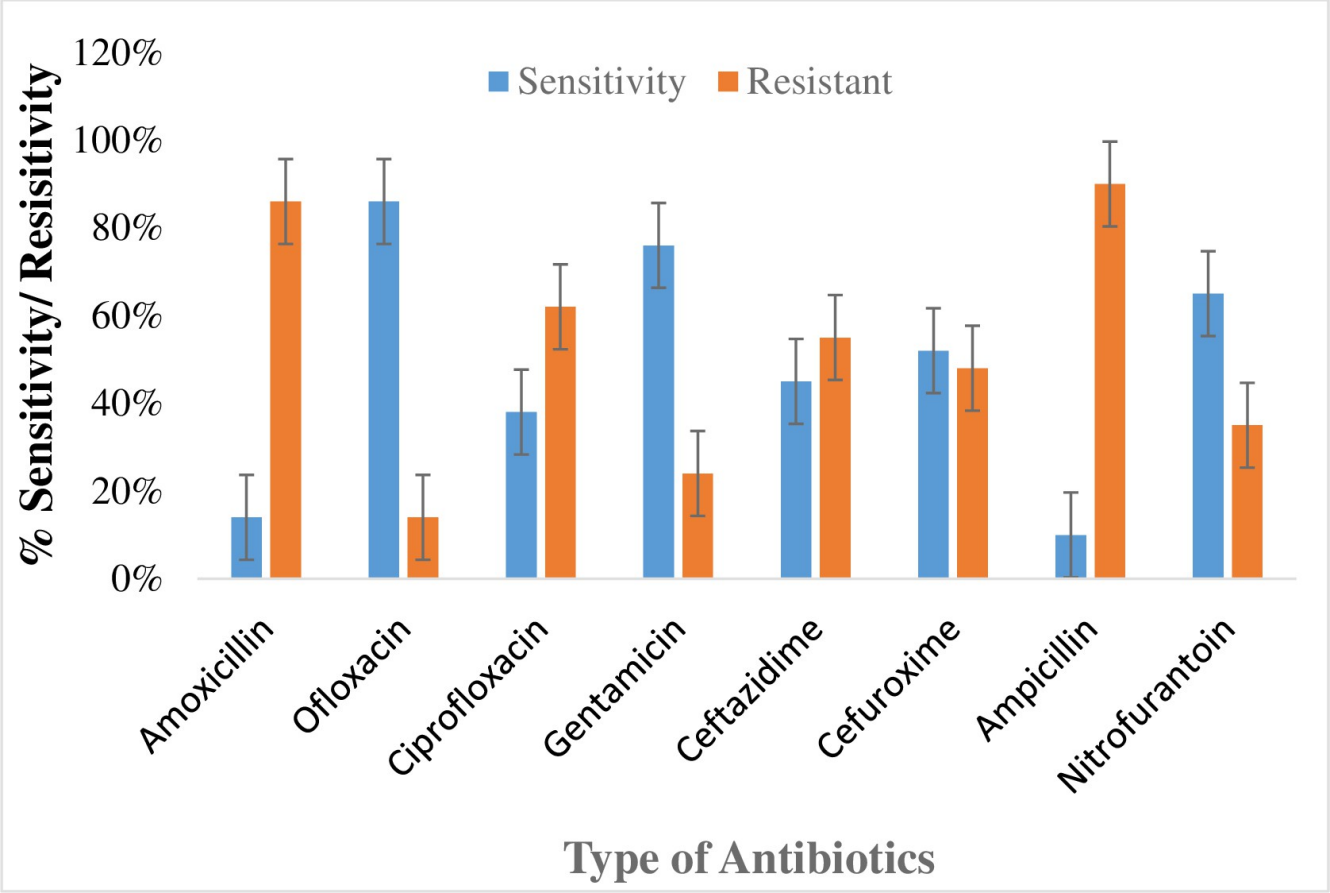
Susceptibility pattern of *E*.*coli* towards antibiotics.

In the study, total 8 antibiotics were checked for susceptibility of isolated bacteria (*E*.*coli*) for sensitivity and resistivity. As a result, *E*.*coli* disclosed highest resistance towards ampicillin 90% and sensitivity 10% while least resistance was observed towards ofloxacin 14% and sensitivity 86%. The result showed resistance towards other antibiotic such as amoxicillin (86%), ciprofloxacin (62%), gentamicin (24%), ceftazidime (55%), cefuroxime (48%) and nitrofurantoin (35%). The sensitivity of *E*.*coli* towards these antibiotics, amoxicillin, ciprofloxacin, gentamicin, ceftazidime, cefuroxime and nitrofurantoin were reported 14, 38, 76, 45, 52 and 65% respectively. The results are summarized as shown in Fig 6.

### Susceptibility pattern of isolated bacteria (*K*. *pneumoniae*) from UTI patients

**Fig 7:**
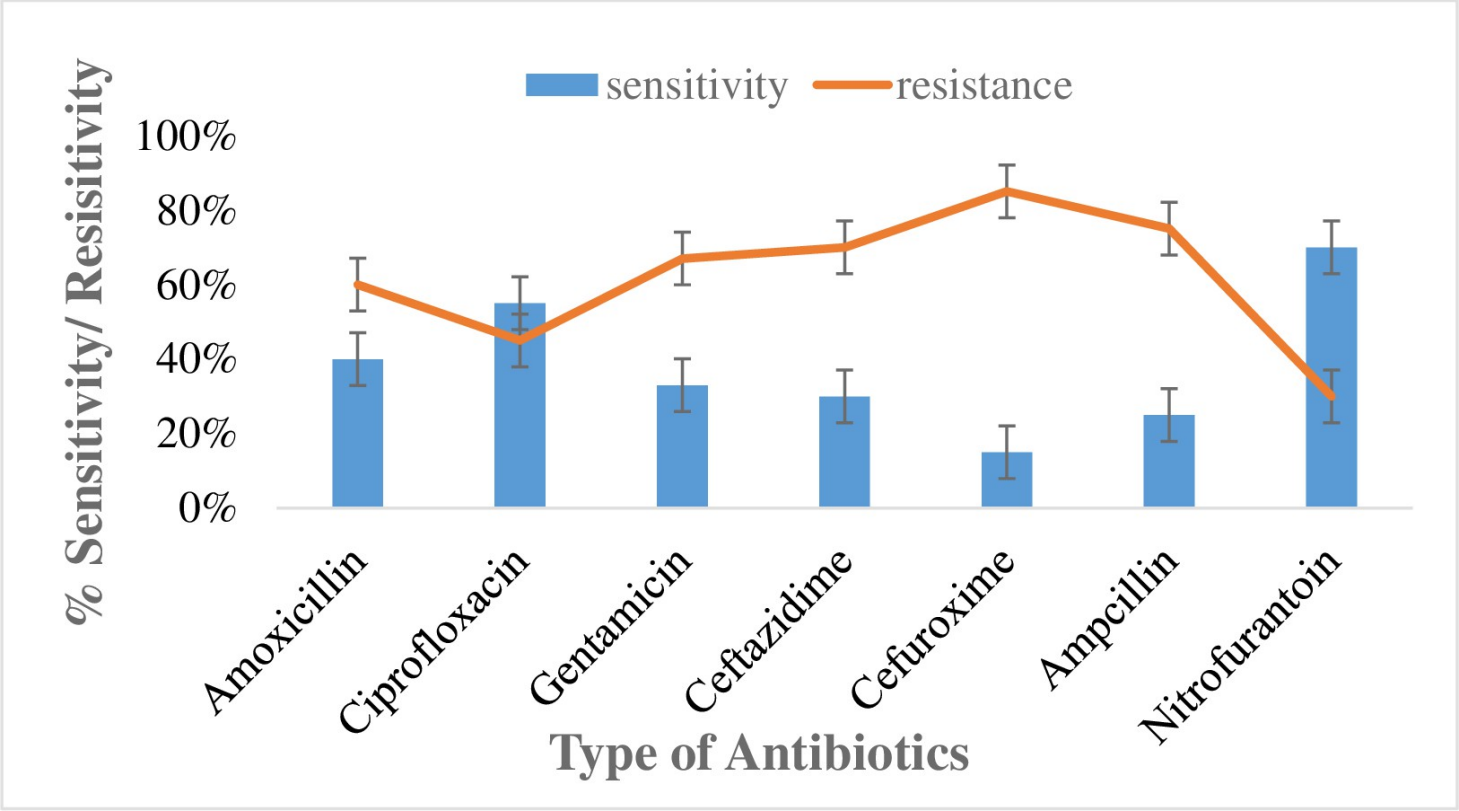
Susceptibility pattern of *K*. *pneumoniae* towards antibiotics.

Antibiotic susceptibility for *k*. *pneumoniae* was reported in which cefuroxime highest antibiotic resistance (85%) against *K*. *pneumoniae* with least sensitivity 15%. The resistance towards amoxicillin, ciprofloxacin, gentamicin, ceftazidime, ampicillin and nitrofurantoin resulted 60, 45, 67, 70, 75 and 30% respectively. The highest sensitivity observed towards nitrofurantoin (70%), followed by amoxicillin (40%), ciprofloxacin (55%), gentamicin (33%), ceftazidime (30%) and ampicillin (25%) as given in the Fig 7.

### Susceptibility pattern of isolated bacteria (*S*. *aureus*) from UTI patients

**Fig 8:**
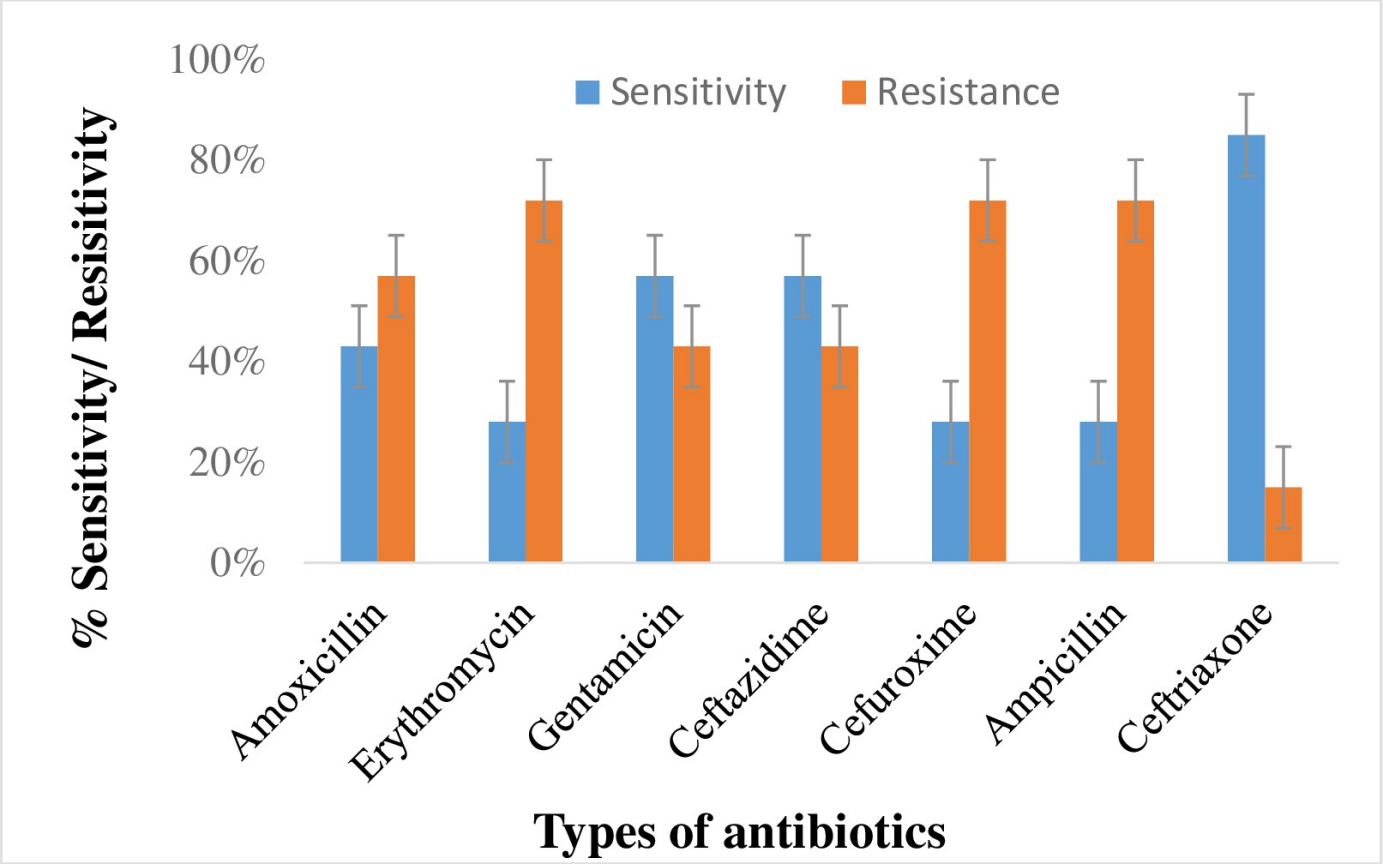
Susceptibility pattern of *S*. *aureus* towards antibiotics.

The sensitivity and resistance of *S*. *aureus* was tested towards 7 different antibiotics, highest resistance was found against erythromycin, cefuroxime and ampicillin which is 72% while 28% sensitivity was observed. The result showed highest sensitivity of *S*. *aureus* to ceftriaxone (85%), followed by amoxicillin (43%), gentamicin (57%) and ceftazidime (57%). The resistance against amoxicillin, gentamicin, ceftazidime and ceftriaxone found were 57, 43, 43 and 15%, respectively as shown in the Fig 8.

## Discussion

Urinary tract infection is a common infection in all age of groups caused by bacteria. The bacteria most responsible causing UTI known as uropathogenic such as *E*. *coli*, *K*. *pneumoniae*, *E*. *faecalis*, *S*. *marcescens*, *P*. *aeruginosa*, *S*. *aureus S*. *saprophyticus*, and *Proteus mirabilis*. Similarly, 50 urine positive sample were collected from both adult male and female genders. Among which 20 positive isolates were from male while 30 from female patients. Our study showed similarities with Asadi Manesh *et al*., [23] that highest prevalence was found in female than male In a relevant study samples screened from UTI patients. In an another study Taye, *et al*., [24] was checked the prevalence on the basis of UTI factors, bacterial isolates and antibiotic susceptibility which is similar to our work.

The outcome of the phylogenetic tree was created with similar sequences by using the neighbor joining method for each isolated bacterial strain from the phylogenetic tree showed that Ai (05) is 99% closely related to *E*. *Coli* (NR 114042.1 *E*.*coli* strain NBRC 102203) Aii (23) is 99% closely related to *K*. *pneumoniae* (NR 117686.1 *K*. *pneumonia* strain DSM 30104). Bi (48) is 96% closely related to *S*. *aureus* (NR 113956.1 *S*. *aureus* strain NBRC 100910). The phylogenetic tree of both studies showed similarities with the study of Mohammed *et al*. [25]. The results of the phylogenetic analysis revealed compatibility values more than 98–99% of proximity and the genetic dimension among themselves in the world.

Phylogenetic trees were created with similar sequences by using BLAST method for each isolated bacterial strain. The phylogenetic tree exposed that Ai (05) is 99.25% closely related to *E*. *coli* (NR 114042.1 *E*. *coli* strain NBRC 102203). Aii (23) is 99% closely related to *K*. *Pneumoniae*, Bi (48) is 96% closely related to *S*. *aureus*. The phylogenetic tree of showed similarities with the previous study [26–28] who find out bacteria in urine samples from UTI patients by 16S rRNA gene sequencing. The outcome of our study was sensitivity and resistance of *S*. *aureus*, tested towards 7 different antibiotics, highest resistance found against erythromycin, cefuroxime and ampicillin. The highest sensitivity of *S*. *aureus* ceftriaxone 85% and displayed highest antibiotic resistance against *K*. *pneumoniae* with least sensitivity in this study. The resistance towards amoxicillin, ciprofloxacin, gentamicin, ceftazidime, ampicillin and nitrofurantoin resulted in 60, 45, 67, 70, 75 and 30% respectively. The highest sensitivity was observed towards nitrofurantoin (70%), followed by amoxicillin (40%), ciprofloxacin (55%), gentamicin (33%), ceftazidime (30%) and ampicillin 25%. Both studies showed variation due to some environmental factors. The find out of our results closely related with the study of Mubanga *et al*. [29] which were study on clinical symptoms of UTI patients through chemistry and microscopy and urine culture. The use of antibacterial drugs are usually helpful to control UTI bacteria but there are also harmful effects which may leads towards serious and chronic conditions of urinary tract infection [30–35].

## Conclusion

The study concludes that participants suffering from UTI showed high growth of *E*. *coli*, followed by *K*. *pneumoniae*, and *S*. *aureus*. Phylogenetic study shows that sequences are closely linked and useful as they provide the similarities and differences among the isolates. The antibiotic susceptibility profiling revealed that *E*. *coli* and *S*. *aureus* are most resistant towards most of the commercially available antibiotics.

## References

[1] Schwartz D. J., Kalas V., Pinkner J. S., Chen S. L., Spaulding C. N., Dodson K. W., et al. (2013). Positively selected FimH residues enhance virulence during urinary tract infection by altering FimH conformation. Proceedings of the National Academy of Sciences, 110(39), 15530–15537. doi: 10.1073/pnas.1315203110 24003161 PMC3785778

[2] Tarchouna M., Ferjani A., Ben-Selma W., & Boukadida J. (2013). Distribution of uropathogenic virulence genes in *Escherichia coli* isolated from patients with urinary tract infection. International Journal of Infectious Diseases, 17(6), e450–e453. doi: 10.1016/j.ijid.2013.01.025 23510539

[3] Manikandan S., Ganesapandian S., Singh M., & Kumaraguru A. K. (2011). Emerging of multidrug resistance human pathogens from urinary tract infections. Current Research in Bacteriology, 4(1), 9–15.

[4] Maharjan G, Khadka P, Siddhi Shilpakar G, Chapagain G, Dhungana GR (2018). Catheter associated urinary tract infection and obstinate biofilm producers. Candian Journal of Infectious Diseases and Medical Microbiology. 1(1):1–7. doi: 10.1155/2018/7624857 30224941 PMC6129315

[5] Sargiary P, Baro L, Choudhry G, Saikia L (2016). Bacteriological profile and antimicrobial susceptibility pattern of community acquired urinary tract infection in children: a tertiary care experience. Journal of Dental and Medical Science. 15(6):61–5.

[6] Aiyegoro O, Igbinosa O, Ogunmwonyi I, Odjadjare E, Igbinosa O, Okoh A (2007). Incidence of urinary tract infections (UTI) among children and adolescents in Ile-Ife, Nigeria. African Journal of Microbiology Research. 1(f2):13–9.

[7] Masika WG, O’Meara WP, Holland TL, Armstrong J (2017). Contribution of urinary tract infection to the burden of febrile illnesses in young children in rural Kenya. PLoS One. 12(3):e0174199. doi: 10.1371/journal.pone.0174199 28323886 PMC5360311

[8] Mitiku E, Amsalu A, Tadesse BT (2018). Pediatric urinary tract infection as a cause of outpatient clinic visits in southern Ethiopia: a cross sectional study. Ethiop J Health Sci.; 28(2):187–96. doi: 10.4314/ejhs.v28i2.10 29983516 PMC6016340

[9] Dhakal BK, Kulesus RR, Mulvey MA (2008) Mechanisms and consequences of bladder cell invasion by uropathogenic *Escherichia coli*. European Journal of Clinical Investigation 38 Suppl 2: 2–11. doi: 10.1111/j.1365-2362.2008.01986.x 18616559

[10] Dashiimaa L, Khosbayar T, Gelegjamts Kh, Malchinkhuu Ch (2010) the clinical features and antibiotic susceptibility of causative bacters of pyelonephritis of children in Mongolia. Journal of Mongolian Medicine 3: 153–155.

[11] Kudinha T, Kong F, Johnson JR, Andrew SD, Anderson P, Gilbert GL (2012) Multiplex PCR-based reverse line blot assay for simultaneous detection of 22 virulence genes in uropathogenic Escherichia coli. Applied Environmental Microbiology 78: 1198–1202. doi: 10.1128/AEM.06921-11 22156422 PMC3272995

[12] Gurevich E., Tchernin D., Schreyber R., Muller R., & Leibovitz E. (2016). Follow-up after infants younger than 2 months of age with urinary tract infection in Southern Israel: epidemiologic, microbiologic and disease recurrence characteristics. Brazilian Journal of Infectious Diseases, 20(1), 19–25. doi: 10.1016/j.bjid.2015.09.003 26607682 PMC9425337

[13] Bliven K. A., & Maurelli A. T. (2016). Evolution of bacterial pathogens within the human host. Microbiology Spectrum, 4(1), 4–1. doi: 10.1128/microbiolspec.VMBF-0017-2015 26999399 PMC4804625

[14] Woo P.C., Lau S.K., Teng J.L., Tse H., Yuen K.Y (2008). Then and now: use of 16S rDNA gene sequencing for bacterial identification and discovery of novel bacteria in clinical microbiology laboratories. Clinical Microbiology Infections. 14 (10):908–934. 10.1111/j.1469-0691.2008.02070.x 18828852

[15] Phumudzo T., Ronald N., Khayalethu N. and Fhatuwani M., 2013. Bacterial species identification getting easier. African Journal of Biotechonlogy, 12: 5975–5982.

[16] Michael J. and Sharon L.A., 2007. 16S rRNA gene sequencing for bacterial identification in the diagnostic laboratory: pluses, perils, and pitfalls. Journal of clinical Microbiology, 45: 2761–2764. doi: 10.1128/JCM.01228-07 17626177 PMC2045242

[17] Badini Z. A., Rauf A., Sanjrani M. A., Niazi M. R., Baseer K., & Khan M. A. (2019). Isolation, Identification, Molecular Characterization and Antibiotic Susceptibility Testing of Uro-Pathogenic E. Coli (UPEC) Isolated From Non-Hospitalized Urinary tract infections (UTI). Pak-Euro Journal of Medical and Life Sciences, 2(4), 69–73.

[18] Khaleque M., Akter S., Akhter H., Khan S. I., & Begum A. (2017). Analysis of diarrheagenic potential of uropathogenic *Escherichia coli* isolates in Dhaka, Bangladesh. The Journal of Infection in Developing Countries, 11(06), 459–469. doi: 10.3855/jidc.8257 30951507

[19] Treves D. S. (2010). Review of three DNA analysis applications for use in the microbiology or genetics classroom. Journal of Microbiology & Biology Education: JMBE, 11(2), 186. doi: 10.1128/jmbe.v11i2.205 23653728 PMC3577175

[20] Olowo-okere A., Ibrahim Y. K. E., Olayinka B. O., Ehinmidu J. O., Mohammed Y., Nabti L. Z., et al. (2020). Phenotypic and genotypic characterization of clinical carbapenem-resistant Enterobacteriaceae isolates from Sokoto, northwest Nigeria. New Microbes and New Infections, 37, 100727. doi: 10.1016/j.nmni.2020.100727 32939286 PMC7479348

[21] Kumar S., Stecher G., & Tamura K. (2016). MEGA7: molecular evolutionary genetics analysis version 7.0 for bigger datasets. Molecular biology and evolution, 33(7), 1870–1874. doi: 10.1093/molbev/msw054 27004904 PMC8210823

[22] Panthi S., Pathak P., & Sitaula J. (2020). Knowledge, attitude and practice on antibiotic use and its resistance among medical students in a tertiary care hospital. Journal of Chitwan Medical College, 10(4), 16–19.

[23] Asadi Manesh F F., Sharifi A., Mohammad Hosini Z., Nasrolahi H., Hosseini N., Kalantari A., et al. (2014). Antibiotic resistance of urinary tract infection of children under 14 years admitted to the pediatric clinic of Imam Sajjad hospital, 2012. Armaghane danesh, 19(5), 411–420.

[24] Taye S., Getachew M., Desalegn Z., Biratu A., & Mubashir K. (2018). Bacterial profile, antibiotic susceptibility pattern and associated factors among pregnant women with Urinary Tract Infection in Goba and Sinana Woredas, Bale Zone, Southeast Ethiopia. BMC Research Notes, 11(1), 1–7. doi: 10.1186/s13104-017-3088-5 30409206 PMC6225670

[25] Mohammed W. S., Ziganshina E. E., Shagimardanova E. I., Gogoleva N. E., & Ziganshin A. M. (2018). Comparison of intestinal bacterial and fungal communities across various xylophagous beetle larvae (Coleoptera: Cerambycidae). Scientific Reports, 8(1), 1–12. doi: 10.1038/s41598-017-17765-5 29968731 PMC6030058

[26] Lakshmi P., Bharadwaj A., & Srivastava R. K. (2020). Molecular Detection and Identification of Bacteria in Urine Samples of Asymptomatic and Symptomatic Pregnant Women by 16S rRNA Gene Sequencing. Archives of Clinical Infectious Diseases, 15(3).

[27] Moustafa A., Li W., Singh H., Moncera K. J., Torralba M. G., Yu Y., et al. (2018). Microbial metagenome of urinary tract infection. Scientific reports, 8(1), 1–12. doi: 10.1038/s41598-017-17765-5 29531289 PMC5847550

[28] Khalid S., Ali Q., Hafeez M., & Malik A. (2021). Perception regarding self-medication of antibiotics in general public sector university of southern Punjab: a comparison between medical and non-medical students. Biological and Clinical Sciences Research Journal, 2021(1). 10.54112/bcsrj.v2021i1.51

[29] Mubanga P., Steinberg W. J., & Van Rooyen F. C. (2015). Antimicrobial susceptibility profile of uropathogens in Maluti Adventist Hospital patients, 2011. African journal of Primary Health Care & Family medicine, 7(1), 1–5. doi: 10.4102/phcfm.v7i1.800 26245603 PMC4683433

[30] Elamary R. B., Albarakaty F. M., & Salem W. M. (2020). Efficacy of Acacia nilotica aqueous extract in treating biofilm-forming and multidrug resistant uropathogens isolated from patients with UTI syndrome. Scientific Reports, 10(1), 1–14. doi: 10.1038/s41598-019-56847-4 32636429 PMC7341837

[31] Horsley H., Dharmasena D., Malone-Lee J., & Rohn J. L. (2018). A urine-dependent human urothelial organoid offers a potential alternative to rodent models of infection. Scientific Reports, 8(1), 1–14. doi: 10.1038/s41598-017-17765-5 29352171 PMC5775255

[32] Nadeem U., Anjum N., Farooq F., Gillani S., & Qurratulain. (2021). A Sonographic evaluation of pediatric acute abdominal pain: A systematic review. Biological and Clinical Sciences Research Journal, 2021(1). 10.54112/bcsrj.v2021i1.60

[33] Yogesha M., Chawla K., Bankapur A., Acharya M., D’Souza J. S., & Chidangil S. (2019). A micro-Raman and chemometric study of urinary tract infection-causing bacterial pathogens in mixed cultures. Analytical and bioanalytical chemistry, 411(14), 3165–3177. doi: 10.1007/s00216-019-01784-4 30989268

[34] Rashid M., Kari M., Rashid R., Rana M., Amjad A., & Hafeez M. (2020). Uterine artery doppler indices as predictive measures for the pre-eclampsia and intrauterine growth restriction. Biological and Clinical Sciences Research Journal, 2020(1). 10.54112/bcsrj.v2020i1.23

[35] Fenta A., Dagnew M., Eshetie S., & Belachew T. (2020). Bacterial profile, antibiotic susceptibility pattern and associated risk factors of urinary tract infection among clinically suspected children attending at Felege-Hiwot comprehensive and specialized hospital, Northwest Ethiopia. A prospective study. BMC Infectious Diseases, 20(1), 1–10. doi: 10.1186/s12879-020-05402-y 32938424 PMC7493977

